# Effect of Statin on Cancer Incidence: An Umbrella Systematic Review and Meta-Analysis

**DOI:** 10.3390/jcm8060819

**Published:** 2019-06-08

**Authors:** Gwang Hun Jeong, Keum Hwa Lee, Jong Yeob Kim, Michael Eisenhut, Andreas Kronbichler, Hans J. van der Vliet, Sung Hwi Hong, Jae Il Shin, Gabriele Gamerith

**Affiliations:** 1 College of Medicine, Gyeongsang National University, Jinju 52727, Korea; gwangh.jeong@gmail.com; 2 Department of Pediatrics, Yonsei University College of Medicine, Yonsei-ro 50, Seodaemun-gu, C.P.O. Box 8044, Seoul 03722, Korea; AZSAGM@yuhs.ac; 3 Division of Pediatric Nephrology, Severance Children’s Hospital, Seoul 03722, Korea; 4 Yonsei University College of Medicine, Seoul 03722, Korea; crossing96@yonsei.ac.kr (J.Y.K.); sunghwihong@gmail.com (S.H.H.); 5 Luton & Dunstable University Hospital NHS Foundation Trust, Lewsey Road, Luton LU4 ODZ, UK; michael_eisenhut@yahoo.com; 6 Department of Internal Medicine IV (Nephrology and Hypertension), Medical University Innsbruck, 6020 Innsbruck, Austria; andreas.kronbichler@i-med.ac.at; 7 Department of Medical Oncology, Amsterdam UMC, Cancer Center Amsterdam, VU University, 1081 HV Amsterdam, The Netherlands; JJ.vanderVliet@vumc.nl; 8 Department of Global Health and Population, Harvard TH Chan School of Public Health, 67 Huntington Avenue, Boston, MA 02115, USA; 9 Institute of Kidney Disease Research, Yonsei University College of Medicine, Seoul 03722, Korea; 10 Department of Medical Oncology, Medical University Innsbruck, 6020 Innsbruck, Austria; gabriele.gamerith@i-med.ac.at

**Keywords:** statin, cancer, meta-analysis, umbrella review

## Abstract

Statins are reported to reduce the risk of cancer, but the results of various published studies have been contradictory. We carried out an umbrella review to provide an overview and understand the strength of evidence, extent of potential biases, and validity of claimed associations between the use of statins and cancer incidence. We comprehensively re-analyzed the data of meta-analyses of randomized controlled trials (RCTs) and observational studies on associations between statin use and cancer incidence. We also assessed the strength of evidence of the re-analyzed outcomes, which were determined from the criteria including statistical significance of the *p*-value of random-effects, as well as fixed-effects meta-analyses, small study effects, between-study heterogeneity, and a 95% prediction interval. Using a conventional method to assess the significance of meta-analysis (*p*-value < 0.05), statins had a statistically significant effect on reducing cancer incidence in 10 of 18 types of cancer. When we graded the level of evidence, no cancer type showed convincing evidence, and four cancers (esophageal cancer, hematological cancer, leukemia, and liver cancer) showed suggestive evidence of a preventive effect. There was weak evidence of an association with six cancers, and no significance for the remaining eight cancers. None of the meta-analyses of RCTs on the association of statin and cancer incidence showed a statistical significance. Although there was a preventive effect of statin on cancer incidence in 10 of the 18 cancer types, the evidence supporting the use of statins to reduce cancer incidence was low. Therefore, the associations between statin use and cancer incidence should be carefully considered by clinicians.

## 1. Introduction

Cancer places one of the biggest burdens on health care system in both highly developed and less developed countries, and its incidence and mortality have been increasing for decades mainly due to longer life expectancy [1]. Based on cancer statistics, 14.1 million new cancers occurred in 2012, while 8.2 million people died of cancer [2]. Despite the efforts of industry and physicians, overall survival and progression-free survival is still unsatisfactory for most cancer types.

Statins, competitively inhibiting 3-hydroxy-3-methylglutaryl coenzyme A reductase, have been used for lowering cholesterol levels [3]. Because of this effect of statin, statins have proven to be effective in reducing the risk of vascular diseases, such as coronary artery disease and stroke [4,5]. Besides their lipid-lowering effects, statins also exhibit anti-inflammatory, immunomodulatory and antithrombotic effects [6,7,8]. It has been proposed that statins also have anti-tumor effects. The mechanism of anti-tumor effect is poorly understood, but some in vitro studies indicate that statins suppress proliferation of tumor cells and angiogenesis [9,10]. Furthermore, epidemiologic studies, clinical trials and meta-analyses also support this benefit in different types of cancer, yet there is a lack of studies, and conflicting results on the relationship between statin use and cancer incidence [11,12,13].

To understand and evaluate the strength of the evidence of the effect of statins on reducing cancer incidence, we carried out an umbrella review and comprehensively re-analyzed the data of meta-analyses of randomized controlled trials (RCTs) and observational studies.

## 2. Materials and Methods

We performed an umbrella review of meta-analyses and systematic reviews reporting on the associations between statin use and the incidence of cancer. This umbrella review was conducted according to the Preferred Reporting Items for Systematic Reviews and Meta-Analyses (PRISMA) guidelines [14]. The PRISMA checklist is shown in the Supplementary Materials.

### 2.1. Literature Search

**Figure 1:**
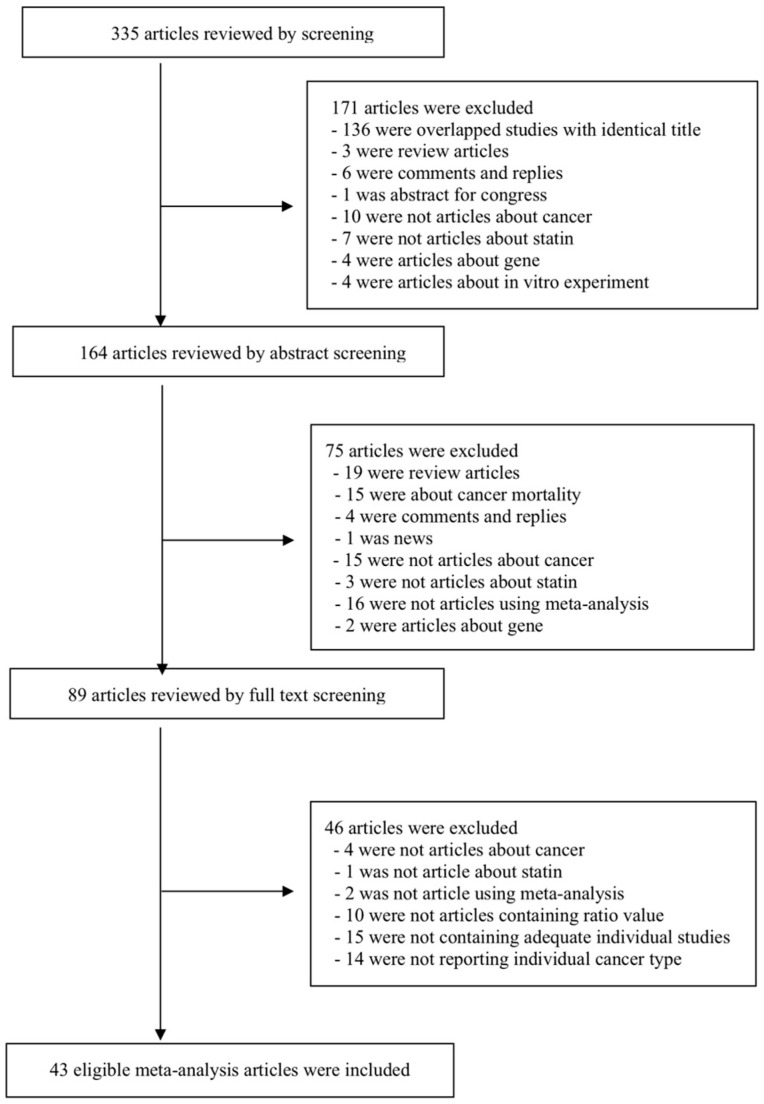
Flow chart of the literature search.

We searched the PubMed database and limited the articles to those written in English, regardless of the publication date. The final search was performed in August 2018. The keywords we used were the following: ‘(hydroxymethyl glutaryl-coa reductase inhibitor OR statin) AND (cancer OR neoplasm OR tumor) AND (meta-analysis OR systematic review)’. Meta analyses of either RCTs or observational studies were included in our search strategy. We reviewed the retrieved articles by examining the titles, the abstracts, and the full texts, then decided which article to include or exclude. We further searched the EMBASE database for potentially eligible meta-analyses, but no additional meta-analysis was included because the identified meta-analyses were lacking data necessary for performing re-analysis or overlapped with the PubMed search. The detailed search strategy is presented in Figure 1.

### 2.2. Eligibility Criteria and Data Extraction

We included meta-analyses and systematic reviews of both RCTs and observational studies reporting on the relationship between statin use and cancer incidence. Observational studies included both cohort and case-control studies. We excluded review articles without meta-analysis, in vitro studies, and genetic studies. We also excluded meta-analyses lacking data necessary for performing re-analysis. If an article presented more than one meta-analysis, all meta-analyses were included and assessed separately by study design or cancer type.

Two investigators (G.H.J. and J.I.S) independently extracted the data, and discrepancies were resolved through consensus. We obtained the data from eligible meta-analyses and extracted and summarized the information on first author, year of publication, the type of cancer, the study design, the number of included studies, the number of cancer cases and total participants, and the random effects with a 95% confidence interval (CI). From the eligible studies, we also extracted the raw data of each individual study for further meta-analysis by combining all data by cancer and study design. If a single study consisted of both RCTs and observational studies, we separated the studies according to the study types (RCTs, observational studies, case-control, and cohort) and reported the results separately.

### 2.3. Statistical Analysis

We firstly re-analyzed each meta-analysis and reported the relationship between statin use and cancer incidence. In addition, if there were overlapping meta-analyses on the same topic, we combined all the individual studies from eligible meta-analyses according to the type of cancer and study design and performed a re-meta-analysis after eliminating overlapping individual studies and including missing individual studies. We presented the summary effect size, 95% CI, and *p*-value with both random- and fixed-effects. All re-analyses in this study were performed using the Comprehensive Meta-Analysis software ver.3.3.070 (Borestein, NH, USA).

### 2.4. Estimation of Summary Effects and Estimation of Prediction Interval

For each meta-analysis, we re-analyzed the individual studies and estimated the summary effects and 95% CI using both random- and fixed-effects methods [15]. We also calculated and presented the 95% prediction interval (PI), which address the dispersion of effects (in 95% of cases the true effect in a new study will fall within the PI) and further account for between-study heterogeneity [16], whereas CI reflects the accuracy of the mean.

### 2.5. Evaluation of Between-Study Heterogeneity and Small Study Effects

We assessed heterogeneity across the studies using the *I*^2^ metric of inconsistency and the *p* value of the *X*^2^-based Cochrane Q test. *I*^2^ values of <50%, 50%–75%, and >75% are usually judged to represent low or moderate, large and very large heterogeneity, respectively [17].

Publication bias was evaluated by using Egger’s regression test [18]. Small study effects were used for detecting publication and reporting bias [19,20]. When the Egger’s test was significant (*p*-value < 0.10) in random-effects meta-analyses, we decided that the study has small-study effects.

### 2.6. Determination of the Level of Evidence

We determined the level of evidence of each meta-analysis and re-analyzed the pooled meta-analysis to classify the strength of the evidence of the association between statin use and cancer incidence. The criteria were set according to the statistical significance by random and fixed-effects *p*-values, 95% PI, a small-study effects, a between-study heterogeneity, and concordance between the effect estimate of the largest study and summary estimate of the meta-analysis [21]. The criteria were as follows:

Convincing evidence: There was a statistical significance for the random-effect and fixed-effect *p*-values at *p <* 0.001. No small study effects or large between-study heterogeneity were found, and 95% PI rejected the null hypothesis. There was a concordance between the effect estimate of the largest study and the summary effect of the random-effects meta-analysis.

Suggestive evidence: There was statistical significance of random effects at *p <* 0.05, but a 95% PI included the null hypothesis. No small study effects or large between-study heterogeneity were found.

Weak evidence: There was a statistical significance of random effects at *p <* 0.05. Small study effects or large between-study heterogeneity were found.

Non-significant association: There was no statistical significance by random effect meta-analysis (*p >* 0.05)

However, if large heterogeneity was found, we rechecked the results to determine whether it might be due to differences in the direction of the effect or if it could be due to differences in the size of the association. In the latter case, we re-determined the level of evidence again.

## 3. Results

### 3.1. Characteristics of Studies Included in the Final Analyses

A total of 335 meta-analyses was retrieved from our PubMed database search, and 43 eligible meta-analyses were selected for re-analysis. At first, 171 articles, including 136 duplicate articles, were excluded by title screening. Another 75 articles were excluded after assessing the abstract, and 46 articles were finally excluded after full text screening. The detailed flow diagram is presented in Figure 1.

Forty-three meta-analyses eligible for our umbrella review investigated the associations between statin use and the incidence of 18 types of cancer [22,23,24,25,26,27,28,29,30,31,32,33,34,35,36,37,38,39,40,41,42,43,44,45,46,47,48,49,50,51,52,53,54,55,56,57,58,59,60,61,62,63,64]. Information on 43 individual meta-analysis is presented in Supplementary Table S1.

### 3.2. Assessing the Effect of Statin on Cancer Incidence with Conventional Interpretation of Meta-Analyses Criteria (Random Effects p-Value < 0.05)

First, we summarized and re-analyzed the results of the previously reported meta-analysis for each stain-cancer incidence association, but there were sometimes discordant results among the meta-analyses of same statin-cancer association. Therefore, we pooled all the individual RCTs and observational studies extracted from eligible studies without missing or overlapping any studies and performed re-meta-analysis in 18 types of cancer to reach a final conclusion of association between statin use and the incidence of one cancer type. Among these, 10 associations (esophageal cancer, hematological cancer, leukemia, liver cancer, breast cancer, colorectal cancer, gastric cancer, lung cancer, lymphoma, and prostate cancer) were statistically significant under the conventional interpretation of meta-analysis criteria (*p <* 0.05), while eight associations (bladder cancer, endometrial cancer, gynecological cancer, kidney cancer, melanoma, myeloma, pancreatic cancer, and non-melanoma skin cancer) were not significant (Table 1).

**Figure 2:**
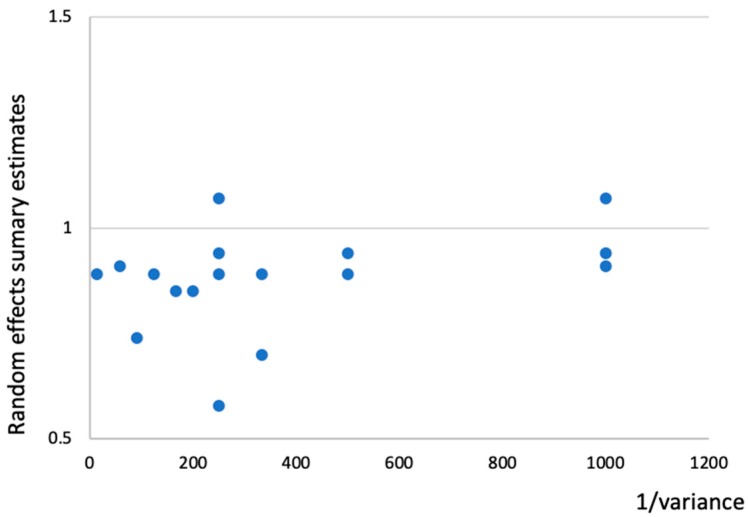
Association of meta-analysis summary effect sizes with the inverse of the variance in cancer incidence.

When associations of the meta-analysis summary effect sizes were analyzed with an inverse of the variance, meta-analyses with small variances showed a trend of summary effects towards 1.00 in cancer incidence, as shown in Figure 2.

### 3.3. Assessing the Statin Effect on Cancer Incidence with Criteria by Previous Umbrella Review

We determined the level of evidence by not only using random effect *p*-values but also by using between-study heterogeneity, small study effects, and 95% PI according to the methods previously published [21]. Under the suggested criteria, we found that none of the associations showed convincing evidence, four associations (esophageal cancer, hematological cancer, leukemia and liver cancer) were found to show suggestive evidence. Six associations (breast cancer, colorectal cancer, gastric cancer, lung cancer, lymphoma, and prostate cancer) showed weak evidence. Details of the graded associations are presented in Table 1.

### 3.4. Re-Analysis of Meta-Analyses Separated by Study Design

In addition to the above process, we performed subgroup analyses of eligible meta-analyses by study designs (RCTs and observational studies) and carried out a re-meta-analysis of the pooled raw data in association with statin use and cancer incidence (Table 2). All overlapping individual studies were omitted while pooling the raw data. Details of the individual overlapping meta-analyses with different study designs on associations with statin and cancer incidence are summarized in Table 3

**Figure 3:**
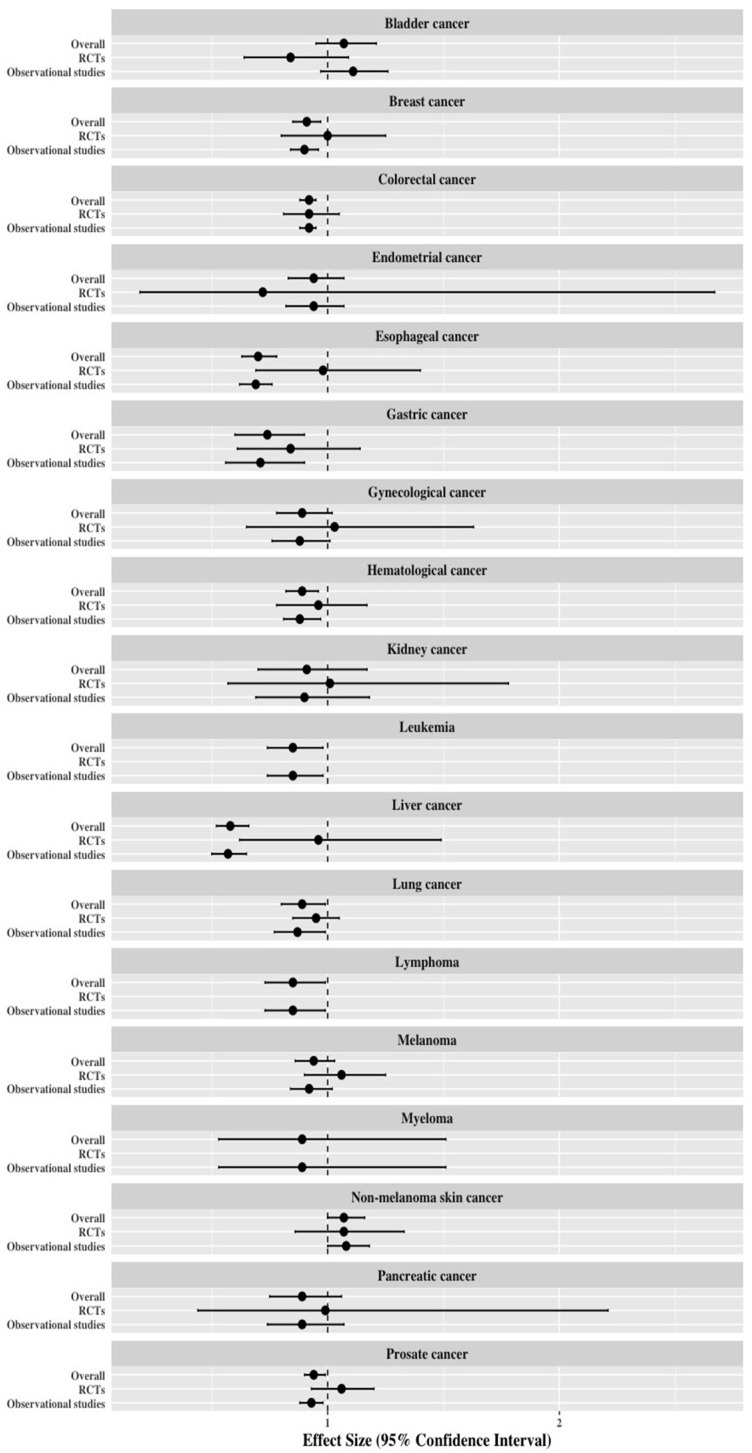
Differences of effect size and 95% confidence interval among the meta-analysis of overall population, randomized controlled trials (RCTs), and observational studies in cancer incidence associated with statin use

Of the 18 types of cancer, three cancer types (leukemia, lymphoma, and myeloma) did not have meta-analyses using RCTs. Among the other 15 cancer types, there was no statistically significant statin–cancer incidence association in meta-analyses of RCTs (Figure 3). For the 18 observational studies, four cancers (esophageal cancer, hematological cancer, leukemia, and liver cancer) showed suggestive evidence, seven cancers (breast cancer, colorectal cancer, gastric cancer, lung cancer, lymphoma, prostate cancer and non-melanoma skin cancer) showed weak evidence, and seven cancers (bladder cancer, endometrial cancer, gynecological cancer, kidney cancer, melanoma, myeloma and pancreatic cancer) were not statistically significant. Therefore, the most significant results of statin-cancer associations were determined by the results of the observational studies.

## 4. Discussion

The purpose of this umbrella review of previous meta-analyses and re-analysis of meta-analyses, including all the individual studies, was to highlight the potential effects of statin use on cancer incidence. We re-analyzed the data from 43 meta-analyses to evaluate the associations between use of statins and cancer incidence. By only using a random-effects *p*-value, 10 of 18 associations of cancer incidence showed a statistically significant preventive effect of statin.

Although there was a weak or non-significant preventive effect of statin use on most cancer types, there was a suggestive level of evidence regarding the preventive effects of statin use on four cancer types (esophageal cancer, hematological cancer, leukemia, and liver cancer). Re-analysis of association between statin use and leukemia incidence was performed with one eligible meta-analysis [31] consisting of nine individual studies, which might be relatively a small number of individual studies for re-analysis. However, associations of the other three cancer types had an adequate number of individual studies (27 for esophageal cancer, 34 for hematological cancer, and 27 for liver cancer). A large number of the included studies for meta-analyses are considered to be valid [65], and, therefore, the outcomes for the 3 cancer types mentioned above might be plausible. Six types of cancer had weak evidence due to substantial publication bias and significant heterogeneity established by the $I^{2}$ value.

Although most of the re-analyses showed weak or non-significant evidence, the conventional interpretation of current meta-analysis is that there was preventive effect of statin use on cancer incidence in some cancer types, based on a random effects *p*-value, an effect size with 95% CI [66]. According to these criteria, 10 of 18 meta-analyses on cancer incidence outcomes demonstrated that statins have a preventive effect on cancer risk.

In addition, while most of the statistically significant individual meta-analyses showed that statins have a preventive effect on cancer, one meta-analysis of observational studies on association with statin and non-melanoma skin cancer suggested that there was a positive relationship between statin and non-melanoma skin cancer [24]. Yang et al. suggested that meta-analyses of observational studies might show more noteworthy result due to the characteristic of observational studies, since it may have advantage of examining rare occurrences of diseases such as cancer. However, the level of evidence in this study was weak, and it included only one meta-analysis. Therefore, we must scrutinize the validity of the results. Further meta-analyses with additional studies will be needed.

Our study evaluates the strength of evidence using multiple values presented or calculated in each meta-analysis. The strength of evidence reinforces the results from the meta-analyses and helps choose the best evidence. Various methods for assessing the evidence level are presented, yet there is no definite grading method for an umbrella review [67,68]. Recent umbrella reviews include the *p*-value of the meta-analysis, between-study heterogeneity, small study effect, and 95% PI for the grading the level of evidence, which is more related to quantitative values [20,21,69].

In addition, substantial heterogeneity is an issue in systematic review and meta-analysis. It is essential to explain and manage the heterogeneity to underline the validity of the respective findings [70]. Umbrella reviews that re-analyze meta-analyses include large number of individual studies, and, therefore, controlling their heterogeneity can be troublesome. Previous umbrella reviews determined a large heterogeneity of $I^{2}$ > 50–75%% as weak evidence [71,72]. However, this application should be applied cautiously, because heterogeneity can increase if the number of individual studies increases. In addition, if the heterogeneity is large, it can be due to differences in the direction of the effect, or it can be due to differences in the size of the association. In the latter case, therefore, we thought the level of evidence should be re-determined and upgraded the level of evidence from weak to suggestive.

In the eligible meta-analyses, overlapping meta-analyses on the same topic were frequently reported (Table 3). Overlapping meta-analyses may give an ambiguous result and should be acknowledged [73]. There are several ways to overcome this problem, and we carried out re-analysis by merging all the extracted individual studies with coherent data. Integration of data from meta-analyses might have more strengths than assembling existing reviews [74]. In our study, the incidence of lymphoma associated with statin use showed a statistically significant outcome with a weak level of evidence, but two other eligible individual meta-analyses of the same association were not significant (Supplementary Table S1). Also, re-analysis of the association of incidence of prostate cancer with statin use was graded as weak evidence, but recent meta-analysis performed by Raval et al. [29] was not significant. Raval et al. only included 27 individual studies, but our study included a total of 64 individual studies, which highlights that there may have been missing eligible studies even in the recent meta-analysis. The comparison of the results of our study and the largest meta-analysis are presented in Table 4.

Results of the meta-analysis can be influenced by study design. Aromataris et al. reported that the types of studies should be matched in systematic reviews, and meta-analyses to be considered for its primary objective [75]. In our study, no meta-analysis that included only RCTs showed a significant preventive effect of statin use on cancer incidence, but re-analyses of observational studies showed statistically significant findings in 11 of the 18 statin–cancer associations. Among these 11 associations, the results of the overall studies (RCTs and observational studies) were determined by those of observational studies in 10 cancers, except non-melanoma skin cancer, for which the results were determined by RCTs. The heterogeneity between overall study design and observational studies may be due to the relatively large number of observational studies included. In addition, observational studies tend to have more biases than RCTs [76], and some reports suggest that the outcomes of observed associations could be false positives or inflated if a large between-study heterogeneity is present [77,78]. However, meta-analysis of RCTs should be interpreted carefully because cancer events are not the primary endpoints of clinical trials. Besides, duration of treatment in clinical trials was relatively shorter than that in observational studies, so there may be an uncertainty of the association.

Our study has several limitations. First, we only assessed individual studies from systematic reviews and meta-analyses eligible for re-analysis, and, therefore, some very recent individual studies might have been missed. However, considering that even a very recent updated meta-analysis for one cancer missed many individual studies, even though thorough search strategy was performed using many search sites such as PubMed, Embase, Scopus, Cochrane database, etc., we think that one should also check the individual studies from previous meta-analyses when updating the meta-analysis. Second, individual studies can have biases, but assessing the quality of individual studies was beyond the scope of our review. Third, exploring the association between dose and types of statins and cancer incidence was also beyond the scope. Likewise, due to a lack of applicable data, we could not stratify the effect of statins by participant age or duration of treatment, which may be the parameter needed to evaluate the true association. Fourth, there were statistical limitations. A 95% PI and Egger *p*-value could not be assessed if there were only two or fewer individual studies. There were also some missing data in the largest study effect when there was no number of population data in individual studies. Finally, 95% PI, between-study heterogeneity, and publication bias may not be definitive criteria for assessing the strength of the evidence.

Nevertheless, in summary, we extensively re-analyzed meta-analyses on the associations between statin use and cancer incidence. In 10 of 18 studies there were significant relationships between statin use and cancer incidence. Although many meta-analyses of RCTs and observational studies reported significant associations between statin use and cancer incidence, only a small portion of these associations were without biases. Also, there was an individual meta-analysis reporting increased risk of cancer associated with statins use, which should be carefully interpreted by researchers and clinicians. Future studies should include more precise individual data, assessment of potential bias, and updated meta-analyses with more qualified RCTs and observational studies. We suggest that clinicians carefully consider the effects of statins on incidence of different types of cancer on the basis of the findings of our study.

## Figures and Tables

**Table 1:** Summary of meta-analyses by combining all the data on associations of the use of statin and the incidence of cancers.

Cancer Type	No of Studies	No of Total Participants	Random Effects (RR, 95%CI)	P (Random)	Fixed Effects (RR, 95%CI)	P (Fixed)	Largest Effect§ (RR, 95%CI)	D/N/I	Egger	*I*^2^ (P) †	95% PI (Random Effects)	95% PI (Fixed Effects)	Small Study Effects	Concordant Direction	Evidence
Bladder cancer	13	1,266,218	1.07 (0.95–1.21)	0.282	1.12 (1.07–1.19)	<0.001	1.08 (0.99–1.19)	0/11/2	0.851	62.6 (0.001)	0.76–1.51	0.81–1.56	No	Yes	Non-significant
Breast cancer	62	3,884,629	0.91 (0.85–0.97)	0.004	1.00 (0.97–1.02)	0.724	1.04 (0.98–1.11)	12/44/3	0.023	79.6 (<0.001)	0.63–1.32	0.69–1.44	Yes	No	Weak
Colorectal cancer	59	13,855,147	0.92 (0.88–0.95)	<0.001	0.94 (0.93–0.96)	<0.001	0.88 (0.81–0.95)	15/33/3	0.106	71.5 (<0.001)	0.76–1.11	0.78–1.14	No	Yes	Weak
Endometrial cancer	15	878,885	0.94 (0.82–1.07)	0.349	1.02 (0.97–1.08)	0.423	1.05 (0.95–1.15)	4/11/0	0.043	54.9 (<0.001)	0.66–1.34	0.73–1.43	Yes	Yes	Non-significant
Esophageal cancer	27	3,158,414	0.70 (0.63–0.78)	<0.001	0.85 (0.71–0.89)	<0.001	0.68 (0.52–0.88)	15/12/0	0.115	60.7 (<0.001)	0.46–1.05	0.50–1.11	No	Yes	Suggestive *
Gastric cancer	16	5,396,224	0.74 (0.60–0.90)	0.004	0.84 (0.79–0.88)	<0.001	0.97 (0.74–1.26)	5/11/0	0.325	90.8 (<0.001)	0.33–1.62	0.39–1.78	No	No	Weak
Gynecological cancer	23	928,721	0.89 (0.78–1.02)	0.087	1.00 (0.93–1.06)	0.899	1.05 (0.95–1.15)	4/19/0	0.003	43.7 (0.014)	0.62–1.29	0.70–1.41	Yes	Yes	Non-significant
Hematological cancer	34	NA	0.89 (0.82–0.96)	0.005	0.86 (0.81–0.90)	<0.001	NA **	7/26/1	0.161	46.7 (0.002)	0.60–1.20	0.64–1.15	No	-	Suggestive
Kidney cancer	11	4,052,120	0.91 (0.70–1.17)	0.457	0.94 (0.88–1.00)	0.034	1.08 (0.99–1.18)	2/9/0	0.722	88.7 (<0.001)	0.39–2.09	0.43–2.05	No	Yes	Non-significant
Leukemia	9	1174	0.85 (0.74–0.98)	0.031	0.83 (0.74–0.92)	0.001	0.74 (0.62–0.87)	2/7/0	0.120	25.0 (0.220)	0.63–1.16	0.62–1.10	No	Yes	Suggestive
Liver cancer	27	2,622,626	0.58 (0.52–0.66)	<0.001	0.65 (0.62–0.68)	<0.001	0.52 (0.41–0.66)	22/5/0	0.117	83.8 (<0.001)	0.33–1.03	0.38–1.13	No	Yes	Suggestive *
Lung cancer	33	8,833,965	0.89 (0.80–0.99)	0.036	0.82 (0.80–0.84)	<0.001	1.03 (0.94–1.21)	5/28/0	0.265	94.9 (<0.001)	0.51–1.57	0.47–1.42	No	No	Weak
Lymphoma	16	8863	0.85 (0.73–0.99)	0.042	0.86 (0.80–0.92)	<0.001	0.96 (0.83–1.11)	6/9/1	0.850	69.1 (<0.001)	0.52–1.40	0.54–1.39	No	No	Weak
Melanoma	24	434,680	0.94 (0.86–1.03)	0.204	0.94 (0.88–1.00)	0.063	0.94 (0.88–1.00)	3/21/0	0.836	26.0 (0.121)	0.74–1.19	0.60–1.46	No	No	Non-significant
Myeloma	5	609	0.89 (0.53–1.51)	0.674	0.89 (0.73–1.09)	0.251	0.83 (0.61–1.12)	2/2/1	0.983	81.0 (<0.001)	0.14–5.73	0.17–4.78	No	Yes	Non-significant
Pancreatic cancer	20	2,832,052	0.89 (0.75–1.06)	0.207	0.91 (0.86–0.97)	0.003	1.10 (0.81–1.49)	1/18/1	0.927	79.0 (<0.001)	0.46–1.71	0.49–1.71	No	Yes	Non-significant
Prostate cancer	44	NA	0.94 (0.90–0.99)	0.017	1.02 (1.00–1.04)	0.056	NA **	18/42/4	0.002	74.5 (<0.001)	0.71–1.24	0.78–1.33	Yes	-	Weak
Non-melanoma skin cancer	17	1,240,281	1.07 (1.00–1.16)	0.063	1.09 (1.06–1.13)	<0.001	1.09 (1.06–1.13)	1/11/5	0.768	58.5 (0.001)	0.88–1.31	0.90–1.32	No	No	Non-significant

D/N/I: Decreasing risk/No difference/Increasing risk; RR: Relative risk; CI: Confidence interval; PI: Prediction interval. § Relative risk (95% Confidence interval) of the largest study in each meta-analysis. † *I***^2^** metric of inconsistency (95% confidence interval of *I***^2^**) and *p*-value of the Cochran Q test for evaluation of heterogeneity. * Suggestive level of evidence due to the greater number of studies that decrease risk in which a high heterogeneity is due to differences in the effect size of the association. ** Largest effect of study of hematologic and prostate cancer were not assessible due to lack of number of participants data in individual studies.

**Table 2:** Re-analysis of the meta-analyses by study design.

Cancer Type	Overall				Randomized Controlled Studies				Observational Studies *			
	No. of Studies	Random Effects (RR, 95%CI)	*p*-Value	Evidence	No. of Studies	Random Effects (RR, 95%CI)	*p*-Value	Evidence	No. of Studies	Random Effects (RR, 95%CI)	*p*-Value	Evidence
Bladder cancer	13	1.07 (0.95–1.21)	0.282	Non-significant	3	0.84 (0.64–1.09)	0.180	Non-significant	10	1.11 (0.97–1.26)	0.118	Non-significant
Breast cancer	62	0.91 (0.85–0.97)	0.004	Weak	12	1.00 (0.80–1.25)	0.661	Non-significant	50	0.90 (0.84–0.96)	0.003	Weak
Colorectal cancer	59	0.92 (0.88–0.95)	<0.001	Weak	13	0.92 (0.81–1.05)	0.214	Non-significant	46	0.92 (0.88–0.95)	<0.001	Weak
Endometrial cancer	15	0.94 (0.83–1.07)	0.349	Non-significant	2	0.72 (0.19–2.67)	0.621	Non-significant	13	0.94 (0.82–1.07)	0.361	Non-significant
Esophageal cancer	27	0.70 (0.63–0.78)	<0.001	Suggestive	1	0.98 (0.69–1.40)	NR	Non-significant	26	0.69 (0.62–0.76)	<0.001	Suggestive
Gastric cancer	16	0.74 (0.60–0.90)	0.004	Weak	3	0.84 (0.61–1.14)	0.259	Non-significant	13	0.71 (0.56–0.90)	0.004	Weak
Gynecological cancer	23	0.89 (0.78–1.02)	0.087	Non-significant	6	1.03 (0.65–1.63)	0.902	Non-significant	17	0.88 (0.76–1.01)	0.069	Non-significant
Hematological cancer	34	0.89 (0.82–0.96)	0.005	Suggestive	8	0.96 (0.78–1.17)	0.667	Non-significant	26	0.88 (0.81–0.97)	0.006	Suggestive
Kidney cancer	11	0.91 (0.70–1.17)	0.457	Non-significant	2	1.01 (0.57–1.78)	0.985	Non-significant	9	0.90 (0.69–1.18)	0.455	Non-significant
Leukemia	9	0.85 (0.74–0.98)	0.031	Suggestive	-	-	-	-	9	0.85 (0.74–0.98)	0.031	Suggestive
Liver cancer	27	0.58 (0.52–0.66)	<0.001	Suggestive	3	0.96 (0.62–1.49)	0.867	Non-significant	24	0.57 (0.50–0.65)	<0.001	Suggestive
Lung cancer	33	0.89 (0.80–0.99)	0.036	Weak	9	0.95 (0.85–1.05)	0.324	Non-significant	24	0.87 (0.77–0.99)	0.034	Weak
Lymphoma	16	0.85 (0.73–0.99)	0.042	Weak	-	-	-	-	16	0.85 (0.73–0.99)	0.042	Weak
Melanoma	24	0.94 (0.86–1.03)	0.204	Non-significant	13	1.06 (0.90–1.25)	0.474	Non-significant	11	0.92 (0.84–1.02)	0.105	Non-significant
Myeloma	5	0.89 (0.53–1.51)	0.674	Non-significant	-	-	-	-	5	0.89 (0.53–1.51)	0.674	Non-significant
Pancreatic cancer	20	0.89 (0.75–1.06)	0.207	Non-significant	3	0.99 (0.44–2.21)	0.982	Non-significant	17	0.89 (0.74–1.07)	0.202	Non-significant
Prostate cancer	64	0.94 (0.90–0.99)	0.017	Weak	7	1.06 (0.93–1.20)	0.386	Non-significant	57	0.93 (0.88–0.98)	0.005	Weak
Non-melanoma skin cancer	17	1.07 (1.00–1.16)	0.063	Non-significant	8	1.07 (0.86–1.33)	0.519	Non-significant	9	1.08 (1.00–1.18)	0.048	Weak

RR: Relative risk. * Observational studies include both cohort studies and case-control studies.

**Table 3:** Summary of individual overlapping meta-analyses with different study designs on associations with statin and cancer incidence.

Cancer Type		Overall				Randomized Controlled Trials				Observational Studies		
		Number of Meta-Analyses	D/N/I	C/S/W		Number of Meta-Analyses	D/N/I	C/S/W		Number of Meta-Analyses	D/N/I	C/S/W
Bladder cancer		1	0/1/0	0/0/0		1	0/1/0	0/0/0		1	0/1/0	0/0/0
Breast cancer		2	0/2/0	0/0/0		1	0/1/0	0/0/0		3	1/2/0	0/1/0
Colorectal cancer		4	4/0/0	1/0/3		4	0/4/0	0/0/0		5	5/0/0	1/0/4
Endometrial cancer		1	0/1/0	0/0/0		1	0/1/0	0/0/0		1	0/1/0	0/0/0
Esophageal cancer		2	2/0/0	1/0/1		0	0/0/0	0/0/0		6	6/0/0	2/4/0
Gastric cancer		2	2/0/0	0/0/2		1	0/1/0	0/0/0		2	2/0/0	0/0/2
Gynecological cancer		1	0/1/0	0/0/0		1	0/1/0	0/0/0		1	0/1/0	0/0/0
Hematological cancer		2	1/1/0	0/0/1		2	0/2/0	0/0/0		2	1/1/0	0/0/1
Kidney cancer		1	0/0/1	0/0/0		1	0/1/0	0/0/0		1	0/1/0	0/0/0
Leukemia		0	0/0/0	0/0/0		0	0/0/0	0/0/0		1	1/0/0	0/1/0
Liver cancer		3	3/0/0	1/1/1		0	0/0/0	0/0/0		1	1/0/0	0/1/0
Lung cancer		2	0/2/0	0/0/0		3	0/3/0	0/0/0		3	0/3/0	0/0/0
Lymphoma		0	0/0/0	0/0/0		0	0/0/0	0/0/0		2	1/1/0	0/0/1
Melanoma		1	0/1/0	0/0/0		3	0/3/0	0/0/0		0	0/0/0	0/0/0
Myeloma		0	0/0/0	0/0/0		0	0/0/0	0/0/0		1	0/1/0	0/0/0
Pancreatic cancer		2	0/2/0	0/0/0		2	0/2/0	0/0/0		2	0/2/0	0/0/0
Prostate cancer		1	0/1/0	0/0/0		1	0/1/0	0/0/0		6	1/5/0	0/0/1
Non-melanoma skin cancer		2	0/1/1	0/0/1		1	0/1/0	0/0/0		1	0/0/1	0/0/1

D/N/I: Decreasing risk/No difference/Increasing risk; C/S/W: Convincing/Suggestive/Weak.

**Table 4:** Comparison of the results with number of included individual studies of our study and the largest meta-analysis.

Type of Cancer		Randomized Controlled Trials						Observational Studies				
		Our Study			Largest Meta-Analysis *			Our Study			Largest Meta-Analysis *	
		No. of Study	Random Effects (RR 95% CI)		No. of Study	Random Effects (RR 95% CI)		No. of Study	Random Effects (RR 95% CI)		No. of Study	Random Effects (RR 95% CI)
Bladder cancer		3	0.84 (0.64–1.09)		3	0.83 (0.64–1.09)		10	1.11 (0.97–1.26)		10	1.11 (0.97–1.26)
Breast cancer		12	1.00 (0.80–1.25)		7	1.19 (0.81–1.73)		50	0.90 (0.84–0.96)		21	0.99 (0.94–1.04)
Colorectal cancer		13	0.92 (0.81–1.05)		11	0.96 (0.85–1.08)		46	0.92 (0.88–0.95)		32	0.92 (0.87–0.96)
Endometrial cancer		2	0.72 (0.19–2.67)		2	0.72 (0.19–2.67)		13	0.94 (0.82–1.07)		13	0.94 (0.82–1.07)
Esophageal cancer		1	0.98 (0.69–1.40)		-	-		26	0.69 (0.62–0.76)		10	0.59 (0.50–0.68)
Gastric cancer		3	0.84 (0.61–1.14)		3	0.84 (0.61–1.14)		13	0.71 (0.56–0.90)		9	0.70 (0.53–0.93)
Gynecological cancer		6	1.03 (0.65–1.63)		6	1.03 (0.65–1.63)		17	0.88 (0.76–1.01)		17	0.88 (0.76–1.01)
Hematological cancer		8	0.96 (0.78–1.17)		6	0.92 (0.78–1.09)		26	0.88 (0.81–0.97)		22	0.88 (0.80–0.98)
Kidney cancer		2	1.01 (0.57–1.78)		2	1.01 (0.57–1.78)		9	0.90 (0.69–1.18)		9	0.90 (0.69–1.18)
Leukemia		-	-		-	-		9	0.85 (0.74–0.98)		9	0.85 (0.74–0.98)
Liver cancer		3	0.96 (0.62–1.49)		-	-		24	0.57 (0.50–0.65)		6	0.58 (0.46–0.74)
Lung cancer		9	0.95 (0.85–1.05)		7	0.95 (0.84–1.09)		24	0.87 (0.77–0.99)		15	0.88 (0.75–1.03)
Lymphoma		-	-		-	-		16	0.85 (0.73–0.99)		13	0.83 (0.69–0.99)
Melanoma		13	1.06 (0.90–1.25)		9	0.92 (0.62–1.36)		11	0.92 (0.84–1.02)		-	-
Myeloma		-	-		-	-		5	0.89 (0.53–1.51)		5	0.89 (0.53–1.51)
Pancreatic cancer		3	0.99 (0.44–2.21)		3	0.99 (0.44–2.21)		17	0.89 (0.74–1.07)		15	0.88 (0.73–1.07)
Prostate cancer		7	1.06 (0.93–1.20)		6	1.06 (0.93–1.20)		57	0.93 (0.88–0.98)		27	0.90 (0.80–1.01)
Non-melanoma skin cancer		8	1.07 (0.86–1.33)		7	1.09 (0.85–1.39)		9	1.08 (1.00–1.18)		5	1.11 (1.02–1.22)

RR: Relative risk; CI: Confidence interval. * Meta-analysis including largest number of individual studies.

## Supplementary Materials

The following are available online at https://www.mdpi.com/2077-0383/8/6/819/s1, Table S1. Reanalysis of each meta-analysis on associations of the use of statin and the incidence of cancers, Table S2. PRISMA checklist
