# Supplementary material for: Effect of Statin on Cancer Incidence: An Umbrella Systematic Review and Meta-Analysis
Source: J Clin Med. 2019 Jun 8;8(6):819. doi: 10.3390/jcm8060819 (PMC6617015; doi:10.3390/jcm8060819)
Supplement: Supplementary file 1 [file jcm-08-00819-s001.pdf]

Supplements

Effect of statin on cancer incidence: an umbrella systematic review and meta-analysis

Supplementary Table S1. Reanalysis of each meta-analysis on associations of the use of statin and the incidence of cancers

Type / Author, year	Study design	No of studies	No of cases/total participants	Random effects (reported) (ES, 95%CI)	Random effects (re-analyzed) (ES, 95%CI)	Fixed effects (re-analyzed) (ES, 95%CI)	Largest effect§	D/N/I	Egger	I^2 (P) †	P (random)	P (fixed)	95% PI (random)	95% PI (fixed)	Small study effects	Concordant direction	Evidence
**Bladder cancer**	Overall	13	466292/1266218	1.07 (0.95-1.21)	1.07 (0.95-1.21)	1.12 (1.07-1.19)	1.08 (0.99-1.19)	0/11/2	0.851	62.6 (0.001)	0.282	<0.001	0.76-1.51	0.81-1.56	No	Yes	Non-significant
Zhang 2013	Overall	13	466292/1266218	1.07 (0.95-1.21)	1.07 (0.95-1.21)	1.12 (1.07-1.19)	1.08 (0.99-1.19)	0/11/2	0.851	62.6 (0.001)	0.282	<0.001	0.76-1.51	0.81-1.56	No	Yes	Non-significant
Zhang 2013	RCT	3	12989/25977	0.83 (0.63-1.10)	0.84 (0.64-1.09)	0.84 (0.64-1.09)	0.81 (0.63-1.10)	0/3/0	0.008	0.0 (0.711)	0.180	0.180	0.15-4.60	0.15-4.60	Yes	Yes	Non-significant
Zhang 2013	Observational studies	10	453303/1240241	-	1.11 (0.97-1.26)	1.14 (1.08-1.21)	1.08 (0.99-1.19)	0/8/2	0.901	66.2 (0.002)	0.118	<0.001	0.77-1.58	0.82-1.59	No	Yes	Non-significant
Zhang 2013	Case-control	5	5268/59847	1.12 (0.98-1.28)	1.12 (0.98-1.28)	1.12 (0.98-1.28)	1.15 (0.94-1.29)	0/5/0	0.983	0.0 (0.681)	0.086	0.086	0.90-1.41	0.90-1.41	No	Yes	Non-significant
Zhang 2013	Cohort	5	448035/1180394	1.11 (0.91-1.35)	1.11 (0.91-1.35)	1.14 (1.08-1.22)	1.08 (0.99-1.19)	0/3/2	0.860	83.5 (<0.001)	0.303	<0.001	0.58-2.13	0.64-2.04	No	Yes	Non-significant
**Breast cancer**	Overall	62	NA/3884629	-	0.91 (0.85-0.97)	1.00 (0.97-1.02)	1.04 (0.98-1.11)	12/44/3	0.023	79.6 (<0.001)	0.004	0.724	0.63-1.32	0.69-1.44	Yes	No	Weak
Islam 2017	Overall	36	121399/2775226	0.95 (0.87-1.04)	0.95 (0.87-1.04)	1.02 (0.99-1.05)	1.01 (0.96-1.06)	3/31/2	0.308	83.8 (<0.001)	0.248	0.195	0.62-1.46	0.66-1.55	No	Yes	Non-significant
Mansourian 2016	Observational studies	16	NA/32445	0.79 (0.74-0.85)	0.77 (0.69-0.86)	0.79 (0.74-0.85)	0.91 (0.78-1.06)	6/10/0	0.084	38.2 (0.061)	<0.001	<0.001	0.57-1.03	0.60-1.05	Yes	No	Suggestive
Undela 2012	Observational studies	21	76759/265482	0.99 (0.94-1.04)	0.99 (0.94-1.04)	1.00 (0.98-1.03)	1.04 (0.98-1.11)	2/18/1	0.288	57.0 (<0.001)	0.695	0.693	0.84-1.16	0.87-1.17	No	Yes	Non-significant
Taylor 2008	Case-control	6	NA/91616	0.86 (0.60-1.23)	0.86 (0.60-1.23)	0.78 (0.67-0.89)	0.90 (0.61-1.32)	1/5/0	0.309	81.6 (<0.001)	0.419	<0.001	0.26-2.87	0.26-2.34	No	Yes	Non-significant
Bonovas 2005	Overall	16	7873/283370	1.02 (0.89-1.18)	1.02 (0.89-1.18)	1.03 (0.93-1.14)	1.02 (0.76-1.36)	1/13/2	0.660	30.0 (0.120)	0.748	0.548	0.72-1.45	0.74-1.44	No	Yes	Non-significant
Bonovas 2005	RCT	7	269/17049	1.19 (0.81-1.73)	1.18 (0.81-1.73)	1.04 (0.81-1.33)	0.75 (0.49-1.13)	0/6/1	0.004	44.0 (0.100)	0.380	0.778	0.45-3.10	0.43-2.52	Yes	Yes	Non-significant
Bonovas 2005	Observational studies	9	7604/266321	1.01 (0.88-1.17)	1.01 (0.88-1.17)	1.03 (0.92-1.15)	1.02 (0.76-1.36)	1/7/1	0.259	26.0 (0.220)	0.874	0.595	0.74-1.39	0.77-1.38	No	Yes	Non-significant
**Colorectal cancer**	Overall	59	NA/13855147	-	0.92 (0.88-0.95)	0.94 (0.93-0.96)	0.88 (0.81-0.95)	15/33/3	0.106	71.5 (<0.001)	<0.001	<0.001	0.76-1.11	0.78-1.14	No	Yes	Weak
Lytras 2014	Overall	40	NA/8302033	-	0.91 (0.87-0.96)	0.94 (0.92-0.96)	0.88 (0.81-0.95)	10/29/1	0.108	70.6 (<0.001)	<0.001	<0.001	0.74-1.13	0.77-1.15	No	Yes	Weak
Lytras 2014	RCT	8	NA/155988	0.89 (0.74-1.07)	0.89 (0.74-1.07)	0.90 (0.78-1.05)	0.87 (0.68-1.12)	0/8/0	0.312	25.0 (0.230)	0.221	0.177	0.60-1.32	0.62-1.31	No	Yes	Non-significant
Lytras 2014	Observational studies	32	13092/8146045	-	0.92 (0.87-0.96)	0.94 (0.92-0.96)	0.88 (0.81-0.95)	10/21/1	0.158	74.8 (<0.001)	<0.001	<0.001	0.74-1.13	0.77-1.16	No	Yes	Weak
Lytras 2014	Case-control	19	100973/1307877	0.92 (0.87-0.98)	0.92 (0.87-0.98)	0.94 (0.91-0.96)	0.92 (0.89-0.96)	6/13/0	0.272	64.0 (<0.001)	0.007	<0.001	0.79-1.09	0.80-1.08	No	Yes	Non-significant
Lytras 2014	Cohort	13	30019/6838168	0.91 (0.83-1.00)	0.91 (0.83-1.00)	0.96 (0.93-0.99)	0.88 (0.81-0.95)	4/8/1	0.221	83.0 (<0.001)	0.042	0.011	0.65-1.26	0.70-1.31	No	Yes	Weak
Liu 2014	Overall	42	NA/7911674	0.90 (0.86-0.95)	0.90 (0.86-0.95)	0.93 (0.91-0.96)	0.91 (0.86-0.96)	9/33/0	0.113	66.5 (<0.001)	<0.001	<0.001	0.71-1.15	0.74-1.18	No	Yes	Weak
Liu 2014	RCT	11	NA/95984	0.96 (0.85-1.08)	0.96 (0.85-1.08)	0.94 (0.86-1.04)	0.90 (0.77-1.03)	0/11/0	0.766	21.6 (0.238)	0.491	0.239	0.74-1.24	0.75-1.20	No	Yes	Non-significant
Liu 2014	Observational studies	31	NA/7911361	-	0.89 (0.94-0.95)	0.93 (0.91-0.96)	0.93 (0.86-1.00)	9/22/0	0.055	72.6 (<0.001)	<0.001	<0.001	0.70-1.14	0.74-1.18	Yes	Yes	Weak
Liu 2014	Case-control	18	NA/344142	0.84 (0.76-0.93)	0.84 (0.76-0.93)	0.93 (0.90-0.96)	0.91 (0.86-0.96)	6/12/0	0.109	78.2 (<0.001)	0.001	<0.001	0.60-1.20	0.67-1.29	No	Yes	Weak
Liu 2014	Cohort	13	NA/7567219	0.93 (0.87-0.99)	0.93 (0.87-0.99)	0.94 (0.91-0.97)	0.93 (0.86-1.00)	3/10/0	0.424	61.6 (0.002)	0.019	0.001	0.76-1.13	0.78-1.13	No	No	Weak
Bardou 2010	Overall	32	NA/1702218	0.92 (0.87-0.97)	0.92 (0.87-0.97)	0.93 (0.91-0.95)	0.92 (0.89-0.96)	7/23/2	0.640	66.0 (<0.001)	0.002	<0.001	0.75-1.13	0.77-1.13	No	Yes	Weak
Bardou 2010	RCT	11	NA/95984	0.93 (0.81-1.08)	0.93 (0.81-1.08)	0.89 (0.81-0.98)	0.79 (0.68-0.91)	1/9/1	0.315	42.0 (0.070)	0.347	0.014	0.65-1.34	0.64-1.25	No	No	Non-significant
Bardou 2010	Observational studies	21	NA/1606234	-	0.92 (0.87-0.98)	0.93 (0.91-0.96)	0.99 (0.92-1.06)	6/14/1	0.569	73.2 (<0.001)	0.006	<0.001	0.75-1.13	0.77-1.14	No	No	Weak
Bardou 2010	Case-control	13	NA/821416	0.92 (0.89-0.95)	0.92 (0.89-0.95)	0.92 (0.90-0.94)	0.92 (0.89-0.96)	4/9/0	0.875	6.0 (0.39)	<0.001	<0.001	NA	NA	No	Yes	-
Bardou 2010	Cohort	8	NA/784818	0.89 (0.75-1.05)	0.89 (0.75-1.05)	0.99 (0.94-1.04)	0.99 (0.92-1.06)	2/5/1	0.315	87.0 (<0.001)	0.166	0.606	0.51-1.55	0.58-1.67	No	Yes	Non-significant
Taylor 2008	Case-control	7	NA/969636	0.89 (0.82-0.97)	0.89 (0.82-0.97)	0.92 (0.89-0.95)	0.94 (0.89-1.00)	2/5/0	0.225	52.8 (0.048)	0.006	<0.001	0.73-1.08	0.78-1.08	No	No	Weak
Bonovas 2007	Overall	18	38374/1553407	0.92 (0.89-0.96)	0.92 (0.89-0.96)	0.92 (0.90-0.95)	0.94 (0.89-1.00)	2/16/0	0.549	7.0 (0.350)	<0.001	<0.001	0.86-0.99	0.86-0.99	No	No	Convincing
Bonovas 2007	RCT	6	610/55113	0.95 (0.80-1.13)	0.95 (0.80-1.13)	0.95 (0.81-1.11)	0.87 (0.68-1.12)	0/6/0	0.878	9.0 (0.360)	0.565	0.518	0.70-1.29	0.71-1.27	No	Yes	Non-significant
Bonovas 2007	Observational studies	12	38124/1498294	0.92 (0.88-0.96)	0.92 (0.88-0.96)	0.92 (0.90-0.95)	0.94 (0.89-1.00)	2/10/0	0.377	16.0(0.290)	<0.001	<0.001	0.86-0.99	0.86-0.99	No	Yes	Convincing
Bonovas 2007	Case-control	9	34054/973545	0.92 (0.89-0.96)	0.91 (0.87-0.96)	0.92 (0.89-0.95)	0.94 (0.89-1.00)	2/7/0	0.268	30.0 (0.180)	0.001	<0.001	0.82-1.02	0.85-0.99	No	No	Suggestive
Bonovas 2007	Cohort	3	4070/498613	0.96 (0.84-1.11)	0.96 (0.84-1.11)	0.96 (0.84-1.11)	0.85 (0.65-1.11)	0/3/0	0.555	0.0 (0.530)	0.594	0.594	0.39-2.36	0.39-2.36	No	Yes	Non-significant
**Endometrial cancer**	Overall	15	NA/878885	0.94 (0.82-1.07)	0.94 (0.83-1.07)	1.02 (0.97-1.08)	1.05 (0.95-1.15)	4/11/0	0.043	54.9 (<0.001)	0.349	0.423	0.66-1.34	0.73-1.43	Yes	Yes	Non-significant
Yang 2017	Overall	15	NA/878885	0.94 (0.82-1.07)	0.94 (0.83-1.07)	1.02 (0.97-1.08)	1.05 (0.95-1.15)	4/11/0	0.043	54.9 (<0.001)	0.349	0.423	0.66-1.34	0.73-1.43	Yes	Yes	Non-significant
Yang 2017	RCT	2	NA/1824	0.72 (0.19-2.67)	0.72 (0.19-2.67)	0.72 (0.19-2.67)	1.03 (0.21-5.08)	0/2/0	-	0.0 (0.43)	0.621	0.621	NA	NA	-	Yes	Non-significant
Yang 2017	Observational studies	13	NA/877061	0.94 (0.82-1.07)	0.94 (0.82-1.07)	1.02 (0.97-1.08)	1.05 (0.95-1.15)	4/9/0	0.054	64.3 (<0.001)	0.361	0.411	0.65-1.37	0.72-1.45	Yes	Yes	Non-significant
**Esophageal caner**	Overall	27	NA/3158414	-	0.70 (0.63-0.78)	0.85 (0.71-0.89)	0.68 (0.52-0.88)	15/12/0	0.115	62.2 (<0.001)	<0.001	<0.001	0.46-1.05	0.50-1.11	No	Yes	Suggestive**
Thomas 2017 (patients with Barett's esophagus)	Observational studies	10	NA/17516	0.59 (0.50-0.68)	0.59 (0.50-0.68)	0.59 (0.50-0.68)	0.61 (0.45-0.83)	7/3/0	0.654	0.0 (0.800)	<0.001	<0.001	0.49-0.70	0.49-0.70	No	Yes	Convincing
Thomas 2017 (esophageal adenocarcinoma)	Observational studies	3	NA/4305	0.57 (0.43-0.76)	0.57 (0.43-0.76)	0.57 (0.43-0.76)	0.58 (0.39-0.87)	2/1/0	0.881	0.0 (0.920)	<0.001	<0.001	0.09-3.76	0.09-3.76	No	Yes	Suggestive
Thomas 2017 (esophageal carcinoma)	Observational studies	7	NA/2042238	0.82 (0.76-0.88)	0.82 (0.76-0.88)	0.82 (0.76-0.88)	0.83 (0.73-0.95)	4/3/0	0.452	0.0 (0.490)	<0.001	<0.001	0.75-0.88	0.75-0.88	No	Yes	Convincing
Beales 2013 (patients with Barett's esophagus)	Observational studies	4	295/1847	0.56 (0.41-0.76)	0.56 (0.42-0.76)	0.56 (0.42-0.76)	0.56 (0.36-0.87)	1/3/0	0.968	0.0 (0.930)	<0.001	<0.001	0.29-1.09	0.29-1.09	No	Yes	Suggestive

Beales 2013 (population-based)	Observational studies	6	NA/2024167	0.81 (0.75-0.88)	0.82 (0.76-0.88)	0.82 (0.76-0.88)	0.84 (0.74-0.96)	4/2/0	0.183	0.0 (0.420)	<0.001	<0.001	0.29-1.09	0.29-1.09	No	Yes	Suggestive
Singh 2013 (esophageal cancer)	Overall	13	9285/1132969	0.72 (0.60-0.86)	0.72 (0.60-0.86)	0.76 (0.70-0.81)	0.44 (0.36-0.53)	6/7/0	0.547	74.0 (<0.001)	<0.001	<0.001	0.40-1.30	0.43-1.33	No	Yes	Weak
Singh 2013 (patients with Barett's esophagus)	Overall	5	312/2125	0.59 (0.45-0.78)	0.59 (0.45-0.78)	0.59 (0.45-0.78)	0.56 (0.36-0.87)	3/2/0	0.254	0.0 (0.540)	<0.001	<0.001	0.38-0.93	0.38-0.93	No	Yes	Convincing
Alexandre 2012 (patients with Barett's esophagus)	Cohort	2	NA/1382	0.53 (0.36-0.78)	0.53 (0.36-0.78)	0.53 (0.36-0.78)	0.56 (0.36-0.87)	2/0/0	-	0.0 (0.666)	0.001	0.001	NA	NA	-	Yes	-
Alexandre 2012 (general population)	Cohort	3	NA/35214	0.86 (0.78-0.94)	0.86 (0.78-0.94)	0.86 (0.78-0.94)	0.84 (0.74-0.96)	1/2/0	0.824	0.0 (0.880)	0.001	0.001	0.49-1.51	0.49-1.51	No	Yes	Suggestive
**Gastric cancer**	Overall	16	NA/5396224	-	0.74 (0.60-0.90)	0.84 (0.79-0.88)	0.97 (0.74-1.26)	5/11/0	0.325	90.8 (<0.001)	0.004	<0.001	0.33-1.62	0.39-1.78	No	No	Weak
Ma 2014	Case-control	6	11056/54800	0.56 (0.35-0.96)	0.56 (0.35-0.89)	0.81 (0.76-0.87)	1.00 (0.89-1.12)	3/3/0	0.274	97.0 (<0.001)	0.015	<0.001	0.11-2.98	0.18-3.62	No	No	Weak
Wu 2013	Overall	12	7611/1002380	0.73 (0.58-0.93)	0.73 (0.58-0.84)	0.79 (0.74-0.84)	0.84 (0.76-0.92)	4/8/0	0.575	88.9 (<0.001)	0.010	<0.001	0.32-1.66	0.36-1.73	No	Yes	Weak
Wu 2013	RCT	3	290/13987	0.84 (0.61-1.14)	0.84 (0.61-1.14)	0.84 (0.67-1.06)	0.92 (0.69-1.22)	1/2/0	0.776	28.5 (0.247)	0.259	0.142	0.05-13.53	0.07-9.70	No	Yes	Non-significant
Wu 2013	Observational studies	9	7321/988393	0.70 (0.53-0.93)	0.70 (0.53-0.93)	0.79 (0.74-0.84)	0.84 (0.76-0.92)	3/6/0	0.477	91.7 (<0.001)	0.013	<0.001	0.27-1.80	0.33-1.90	No	Yes	Weak
Singh 2013	Overall	11	5581/5459975	0.68 (0.51-0.91)	0.68 (0.51-0.91)	0.76 (0.71-0.82)	0.97 (0.74-1.26)	4/7/0	0.496	89.0 (<0.001)	0.010	<0.001	0.25-1.83	0.30-1.94	No	No	Weak
**Gynecological cancer**	Overall	24(23)*	12904/928721	0.89 (0.78-1.01)	0.89 (0.78-1.02)	1.00 (0.93-1.06)	1.05 (0.95-1.15)	4/20(19)/0	0.003	43.7 (0.014)	0.087	0.899	0.62-1.29	0.70-1.41	Yes	Yes	Non-significant
Liu 2014	Overall	24(23)*	12904/928721	0.89 (0.78-1.01)	0.89 (0.78-1.02)	1.00 (0.93-1.06)	1.05 (0.95-1.15)	4/20(19)/0	0.003	43.7 (0.014)	0.087	0.899	0.62-1.29	0.70-1.41	Yes	Yes	Non-significant
Liu 2014	RCT	7(6)*	78/2252	0.97 (0.62-1.50)	1.03 (0.65-1.63)	1.03 (0.65-1.63)	0.97 (0.62-1.50)	0/6/0	0.031	0.0 (0.581)	0.902	0.902	0.54-1.97	0.54-1.97	Yes	Yes	Non-significant
Liu 2014	Observational studies	17	12826/916459	-	0.88 (0.76-1.01)	1.00 (0.93-1.06)	1.05 (0.95-1.15)	4/13/0	0.005	54.6 (0.004)	0.069	0.884	0.59-1.32	0.68-1.46	Yes	Yes	Non-significant
Liu 2014	Case-control	7	7675/36385	0.61 (0.40-0.91)	0.61 (0.40-0.91)	0.62 (0.46-0.84)	0.48 (0.26-0.89)	3/4/0	0.518	41.7 (0.113)	0.015	0.002	0.22-1.70	0.24-1.65	No	Yes	Suggestive
Liu 2014	Cohort	10	5151/880074	0.97 (0.87-1.09)	0.97 (0.87-1.09)	1.02 (0.95-1.09)	1.05 (0.95-1.15)	1/9/0	0.101	40.0 (0.091)	0.647	0.572	0.74-1.28	0.79-1.31	No	Yes	Non-significant
**Hematological cancer**	Overall	35(34) *	NA	-	0.89 (0.82-0.96)	0.86 (0.81-0.90)	-	8(7)/26/1	0.161	46.7 (0.002)	0.005	<0.001	0.60-1.20	0.64-1.15	No	-	Suggestive
Pradelli 2015	Observational studies	23(22)*	14442/NA	0.86 (0.77-0.96)	0.88 (0.80-0.98)	0.85 (0.80-0.90)	0.78 (0.71-0.86)	7(6)/15/1	0.281	55.6 (0.001)	0.018	<0.001	0.62-1.25	0.61-1.19	No	Yes	Weak
Pradelli 2015	Case-control	11(10)*	NA/11560	0.83 (0.62-1.09)	0.89 (0.65-1.20)	0.80 (0.73-0.87)	0.78 (0.71-0.86)	5(4)/5/1	0.570	74.6 (<0.001)	0.443	<0.001	0.35-2.25	0.34-1.89	No	No	Non-significant
Pradelli 2015	Cohort	12	NA/2882	0.89 (0.82-0.95)	0.89 (0.82-0.95)	0.89 (0.82-0.95)	0.84 (0.72-0.98)	2/10/0	0.516	0.0 (0.660)	0.001	0.001	0.92-0.95	0.82-0.95	No	Yes	Convincing
Yi 2014	Overall	20(19)*	15297/1139584	0.81 (0.70-0.92)	0.83 (0.73-0.95)	0.81 (0.75-0.86)	0.78 (0.71-0.86)	6(5)/13/1	0.609	54.2 (0.003)	0.008	<0.001	0.55-1.26	0.55-1.19	No	Yes	Weak
Yi 2014	RCT	6	522/45526	0.92 (0.77-1.09)	0.92 (0.78-1.09)	0.92 (0.78-1.09)	1.01 (0.81-1.25)	0/6/0	0.330	0.0 (0.560)	0.344	0.344	0.34-0.56	0.34-0.56	No	Yes	Non-significant
Yi 2014	Observational studies	14(13)*	14775/1094058	0.79 (0.67-0.93)	0.82 (0.70-0.97)	0.79 (0.73-0.85)	0.78 (0.71-0.86)	6(5)/7/1	0.635	63.3 (0.001)	0.021	<0.001	0.51-1.34	0.50-1.24	No	Yes	Weak
Bonovas 2007	Overall	14(13)*	5629/412053	0.85 (0.64-1.12)	0.91 (0.68-1.20)	0.90 (0.78-1.04)	0.88 (0.60-1.29)	3(2)/10/1	0.983	67.8 (<0.001)	0.495	0.168	0.55-1.26	0.55-1.19	No	Yes	Non-significant
Bonovas 2007	RCT	6	311/46852	0.92 (0.72.1.16)	0.92 (0.70-1.21)	0.94 (0.75-1.18)	1.32 (0.85-1.77)	0/6/0	0.453	6.0 (0.380)	0.542	0.603	0.72-1.18	0.72-1.18	No	Yes	Non-significant
Bonvoas 2007	Observational studies	8(7)*	5308/365201	0.83 (0.53-1.29)	0.91 (0.55-1.51)	0.87 (0.72-1.06)	0.88 (0.60-1.29)	3(2)/4/1	0.825	80.3 (<0.001)	0.714	0.171	0.51-1.34	0.50-1.24	No	Yes	Non-significant
**Kidney cancer**	Overall	12(11)*	954880/4052120	0.92 (0.71-1.19)	0.91 (0.70-1.17)	0.94 (0.88-1.00)	1.08 (0.99-1.18)	2/10(9)/0	0.722	88.7 (<0.001)	0.457	0.034	0.39-2.09	0.43-2.05	No	Yes	Non-significant
Zhang 2014	Overall	12(11)*	954880/4052120	0.92 (0.71-1.19)	0.91 (0.70-1.17)	0.94 (0.88-1.00)	1.08 (0.99-1.18)	2/10(9)/0	0.722	88.7 (<0.001)	0.457	0.034	0.39-2.09	0.43-2.05	No	Yes	Non-significant
Zhang 2014	RCT	2	10768/21533	1.01 (0.57-1.79)	1.01 (0.57-1.78)	1.01 (0.57-1.78)	1.04 (0.58-1.86)	0/2/0	-	87.8 (<0.001)	0.985	0.985	NA	NA	-	Yes	Non-significant
Zhang 2014	Observational studies	9	944112/4030587	-	0.90 (0.69-1.18)	0.93 (0.88-1.00)	1.08 (0.99-1.18)	2/8(7)/0	0.743	90.9 (<0.001)	0.455	0.033	0.37-2.20	0.37-2.37	No	Yes	Non-significant
Zhang 2014	Case-control	5	1989/520704	0.74 (0.45-1.23)	0.74 (0.45-1.23)	0.58 (0.51-0.67)	0.52 (0.45-0.60)	2/3/0	0.395	78.6 (0.001)	0.244	<0.001	0.14-3.92	0.14-2.53	No	No	Non-significant
Zhang 2014	Cohort	5(4)*	942123/3509883	1.07 (0.96-1.20)	1.07 (0.96-1.19)	1.08 (1.00-1.16)	1.08 (0.99-1.18)	0/5(4)/0	0.643	30.6 (0.217)	0.210	0.041	0.75-1.53	0.80-1.46	No	Yes	Non-significant
**Leukemia**	Observational studies	9	NA/1174	0.83 (0.74-0.92)	0.85 (0.74-0.98)	0.83 (0.74-0.92)	0.74 (0.62-0.87)	2/7/0	0.120	25.0 (0.220)	0.031	0.001	0.63-1.16	0.62-1.10	No	Yes	Suggestive
Pradelli 2015	Observational studies	9	NA/1174	0.83 (0.74-0.92)	0.85 (0.74-0.98)	0.83 (0.74-0.92)	0.74 (0.62-0.87)	2/7/0	0.120	25.0 (0.220)	0.031	0.001	0.63-1.16	0.62-1.10	No	Yes	Suggestive
**Liver cancer**	Overall	27	NA/2622626	-	0.58 (0.52-0.66)	0.65 (0.62-0.68)	0.52 (0.41-0.66)	22/5/0	0.117	83.8 (<0.001)	<0.001	<0.001	0.33-1.03	0.38-1.13	No	Yes	Suggestive**
Zhong 2016	Overall	24	NA/2167078	0.60 (0.52-0.69)	0.60 (0.53-0.69)	0.66 (0.63-0.68)	0.53 (0.49-0.58)	19/5/0	0.239	85.0 (<0.001)	<0.001	<0.001	0.34-1.06	0.38-1.14	No	Yes	Suggestive**
Shi 2014	Overall	13	35756/5640313	0.58 (0.51-0.67)	0.58 (0.51-0.67)	0.57 (0.54-0.61)	0.53 (0.49-0.58)	11/2/0	0.729	65.0 (<0.001)	<0.001	<0.001	0.38-0.89	0.39-0.85	No	Yes	Convincing**
Singh 2013	Overall	11	4298/1459417	0.63 (0.52-0.76)	0.63 (0.52-0.76)	0.64 (0.58-0.71)	0.52 (0.41-0.66)	6/5/0	0.879	59.0 (0.010)	<0.001	<0.001	0.37-1.07	0.39-1.05	No	Yes	Weak
Pradelli 2013	Observational studies	6	2574/738873	0.58 (0.46-0.74)	0.58 (0.46-0.74)	0.63 (0.56-0.71)	0.74 (0.64-0.87)	5/1/0	0.451	65.0 (0.010)	<0.001	<0.001	0.29-1.16	0.34-1.18	No	Yes	Suggestive**
**Lung cancer**	Overall	33	NA/8833965	-	0.89 (0.80-0.99)	0.82 (0.80-0.84)	1.03 (0.94-1.21)	5/28/0	0.265	94.9 (<0.001)	0.036	<0.001	0.51-1.57	0.47-1.42	No	No	Weak
Wang 2013	Overall	20	37560/4980009	0.89 (0.78-1.02)	0.89 (0.78-1.02)	0.85 (0.82-0.87)	0.81 (0.77-0.86)	3/17/0	0.589	93.6 (<0.001)	0.103	<0.001	0.51-1.56	0.49-1.45	No	No	Non-significant
Wang 2013	RCT	5	14830/29658	0.92 (0.79-1.06)	0.92 (0.79-1.06)	0.92 (0.79-1.06)	0.93 (0.76-1.09)	0/5/0	0.483	0.0 (<0.636)	0.248	0.248	0.72-1.17	0.72-1.17	No	Yes	Non-significant
Wang 2013	Observational studies	15	734373/4145990	-	0.88 (0.75-1.03)	0.84 (0.82-0.87)	0.81 (0.77-0.86)	3/12/0	0.705	95.2 (<0.001)	0.113	<0.001	0.49-1.60	0.48-1.49	No	No	Non-significant
Wang 2013	Case-control	7	19328/575804	0.81 (0.57-1.16)	0.82 (0.57-1.16)	0.72 (0.68-0.75)	0.55 (0.52-0.59)	1/6/0	0.664	96.4 (<0.001)	0.252	<0.001	0.25-2.68	0.32-2.91	No	No	Non-significant
Wang 2013	Cohort	8	712045/3570186	0.93 (0.82-1.06)	0.93 (0.82-1.06)	0.92 (0.89-0.95)	0.81 (0.77-0.86)	2/6/0	0.818	87.8 (<0.001)	0.277	<0.001	0.62-1.39	0.63-1.33	No	No	Non-significant
Tan 2013	Overall	19	52388/5008404	-	0.89 (0.77-1.03)	0.82 (0.80-0.84)	0.55 (0.52-0.59)	3/16/0	0.337	93.3 (<0.001)	0.117	<0.001	0.50-1.60	0.47-1.45	No	No	Non-significant
Tan 2013	RCT	5	14830/29658	0.91 (0.76-1.09)	0.91 (0.76-1.09)	0.91 (0.76-1.08)	0.81 (0.58-1.13)	0/5/0	0.293	0.0 (0.633)	0.311	0.311	0.67-1.23	0.67-1.23	No	Yes	Non-significant
Tan 2013	Observational studies	14	37558/4979746	0.88 (0.75-1.04)	0.88 (0.75-1.04)	0.82 (0.79-0.84)	0.55 (0.52-0.59)	3/11/0	0.433	95.1 (<0.001)	0.124	<0.001	0.47-1.65	0.45-1.49	No	No	Non-significant
Tan 2013	Case-control	7	18978/988565	0.81 (0.57-1.16)	0.82 (0.57-1.16)	0.72 (0.68-0.75)	0.55 (0.52-0.59)	1/6/0	0.665	96.4 (<0.001)	0.253	<0.001	0.25-2.68	0.24-2.17	No	No	Non-significant
Tan 2013	Cohort	7	18580/3991181	0.94 (0.82-1.07)	0.94 (0.82-1.07)	0.89 (0.86-0.93)	0.81 (0.77-0.86)	2/5/0	0.395	87.8 (<0.001)	0.326	<0.001	0.60-1.46	0.59-1.34	No	No	Non-significant

Taylor 2008	Case-control	4	NA/578428	0.75 (0.50-1.11)	0.75 (0.50-1.11)	0.53 (0.50-0.56)	0.52 (0.49-0.55)	1/3/0	0.039	79.3 (0.002)	0.152	<0.001	0.13-4.20	0.12-2.36	Yes	No	Non-significant
Bonovas 2006	RCT	7	74/63353	0.95 (0.83-1.09)	0.95 (0.84-1.09)	0.95 (0.84-1.09)	1.07 (0.87-1.32)	0/7/0	0.805	0.0 (0.420)	0.477	0.477	0.79-1.14	0.79-1.14	No	Yes	Non-significant
**Lymphoma**	Observational studies	17(16)*	NA/8863	-	0.85 (0.73-0.99)	0.86 (0.80-0.92)	0.96 (0.83-1.11)	7(6)/9/1	0.850	69.1 (<0.001)	0.042	<0.001	0.52-1.40	0.54-1.39	No	No	Weak
Ye 2017	Observational studies	13	NA/7825	0.82 (0.69-0.99)	0.83 (0.69-0.99)	0.86 (0.80-0.93)	0.96 (0.83-1.11)	5/7/1	0.734	72.9 (<0.001)	0.041	<0.001	0.46-1.47	0.50-1.49	No	No	Weak
Pradelli 2015	Observational studies	10(9)*	NA/3469	0.81 (0.68-0.96)	0.86 (0.74-1.00)	0.87 (0.80-0.96)	0.94 (0.80-1.11)	4(3)/6/0	0.810	42.5 (0.084)	0.051	0.003	0.60-1.23	0.63-1.21	No	No	Non-significant
**Melanoma**	Overall	24	NA/434680	-	0.94 (0.86-1.03)	0.94 (0.88-1.00)	0.79 (0.66-0.96)	3/21/0	0.836	26.0 (0.121)	0.204	0.063	0.75-1.19	0.76-1.17	No	No	Non-significant
Li 2014	Overall	21	8433/529335	0.94 (0.85-1.04)	0.94 (0.86-1.03)	0.94 (0.88-1.00)	0.79 (0.66-0.96)	3/18/0	0.959	33.8 (0.070)	0.182	0.052	0.74-1.19	0.60-1.46	No	No	Non-significant
Bonovas 2010	RCT	9	NA/62658	0.92 (0.62-1.36)	0.92 (0.62-1.36)	0.93 (0.68-1.27)	1.70 (0.78-3.71)	1/8/0	0.680	22.0 (0.470)	0.686	0.639	0.42-2.02	0.45-1.93	No	Yes	Non-significant
Freeman 2006	RCT	6	154/35088	0.87 (0.61-1.23)	0.88 (0.57-1.34)	0.87 (0.61-1.25)	1.07 (0.64-1.79)	1/5/0	0.907	16.6 (0.310)	0.538	0.457	0.38-2.02	0.40-1.89	No	Yes	Non-significant
Dellavalle 2005	RCT	5	126/31198	0.90 (0.56-1.44)	0.90 (0.56-1.44)	0.88 (0.62-1.26)	1.07 (0.64-1.79)	1/4/0	0.638	60.0 (0.080)	0.651	0.497	0.27-2.92	0.30-2.57	No	Yes	Non-significant
**Myeloma**	Observational studies	5	NA/609	0.89 (0.53-1.51)	0.89 (0.53-1.51)	0.89 (0.73-1.09)	0.83 (0.61-1.12)	2/2/1	0.983	81.0 (<0.001)	0.674	0.251	0.14-5.73	0.17-4.78	No	Yes	Non-significant
Pradelli 2015	Observational studies	5	NA/609	0.89 (0.53-1.51)	0.89 (0.53-1.51)	0.89 (0.73-1.09)	0.83 (0.61-1.12)	2/2/1	0.983	81.0 (<0.001)	0.674	0.251	0.14-5.73	0.17-4.78	No	Yes	Non-significant
**Pancreatic cancer**	Overall	21(20)*	NA/2832052	-	0.89 (0.75-1.06)	0.91 (0.86-0.97)	1.10 (0.81-1.49)	1/19(18)/1	0.927	79.0 (<0.001)	0.207	0.003	0.46-1.71	0.49-1.71	No	Yes	Non-significant
Cui 2012	Overall	18	7807/1692863	0.89 (0.74-1.07)	0.89 (0.74-1.07)	0.91 (0.86-0.97)	0.95 (0.87-1.04)	1/16/1	0.889	37.7 (0.200)	0.216	0.003	0.46-1.74	0.48-1.74	No	Yes	Non-significant
Cui 2012	RCT	3	NA/7118	0.99 (0.44-2.21)	0.99 (0.44-2.21)	0.99 (0.44-2.21)	0.90 (0.37-2.20)	0/3/0	0.494	0.0 (0.840)	0.982	0.982	0.01-178.30	0.01-178.30	No	Yes	Non-significant
Cui 2012	Observational studies	15	NA/3166078	-	0.88 (0.73-1.07)	0.91 (0.86-0.97)	0.93 (0.77-1.12)	1/13/1	0.803	84.3 (<0.001)	0.211	0.003	0.44-1.78	0.47-1.78	No	Yes	Non-significant
Cui 2012	Case-control	8	4836/542048	0.74 (0.51-1.07)	0.74 (0.51-1.07)	0.70 (0.62-0.79)	0.93 (0.77-1.12)	1/7/0	0.781	86.0 (<0.001)	0.108	<0.001	0.21-2.55	0.22-2.23	No	Yes	Non-significant
Cui 2012	Cohort	7	NA	1.05 (0.93-1.19)	1.05 (0.93-1.18)	1.01 (0.94-1.08)	0.95 (0.87-1.04)	0/6/1	0.287	40.0 (0.130)	0.402	0.818	0.78-1.41	0.78-1.30	No	Yes	Non-significant
Bonovas 2008	Overall	13(11)*	3521/914601	0.88 (0.63-1.23)	0.86 (0.61-1.21)	0.91 (0.84-0.99)	0.37 (0.30-0.46)	1/12(10)/0	0.788	87.8 (<0.001)	0.379	0.022	0.30-2.47	0.34-2.44	No	No	Non-significant
Bonovas 2008	RCT	3	NA/7118	0.99 (0.44-2.21)	0.99 (0.44-2.21)	0.99 (0.44-2.21)	0.90 (0.37-2.21)	0/3/0	0.493	0.0 (0.835)	0.983	0.983	0.01-178.30	0.01-178.30	No	Yes	Non-significant
Bonovas 2008	Observational studies	10(8)*	3515/907843	0.86 (0.60-1.24)	0.84 (0.58-1.22)	0.91 (0.84-0.99)	0.37 (0.30-1.46)	1/7/0	0.705	91.4 (<0.001)	0.351	0.022	0.26-2.74	0.30-2.72	No	Yes	Non-significant
Bonovas 2008	Case-control	5	3301/542734	0.70 (0.37-1.33)	0.70 (0.37-1.33)	0.84 (0.77-0.93)	0.37 (0.30-0.46)	1/4/0	0.597	95.0 (<0.001)	0.279	<0.001	0.07-7.20	0.10-6.79	No	No	Non-significant
Bonovas 2008	Cohort	5(3)*	214/364749	1.11 (0.95-1.29)	1.1 (0.95-1.29)	1.1 (0.95-1.29)	1.09 (0.89-1.34)	0/3/0	0.182	0.0 (0.950)	0.193	0.193	0.41-2.96	0.41-2.96	No	Yes	Non-significant
**Prostate cancer**	Overall	67(64)*	NA	-	0.94 (0.90-0.99)	1.02 (1.00-1.04)	-	19(18)/44 (42)/4	0.002	74.5 (<0.001)	0.017	0.056	0.71-1.24	0.78-1.33	Yes	-	Weak
Raval 2016	Cohort	27	NA	0.90 (0.80-1.01)	0.90 (0.80-1.01)	0.90 (0.84-0.96)	0.88 (0.76-1.02)	9/16/2	0.938	62.0 (<0.001)	0.067	0.001	0.56-1.43	0.57-1.42	No	Yes	Non-significant
Scosyrev 2013	Cohort	8	2812/12843	0.91 (0.72-1.13)	0.90 (0.72-1.13)	0.93 (0.83-1.04)	0.99 (0.83-1.18)	3/4/1	0.679	68.7 (0.002)	0.382	0.197	0.45-1.80	0.49-1.77	No	Yes	Non-significant
Bansal 2012	Observational studies	26(25)*	56845/1893356	0.93 (0.87-0.99)	0.93 (0.86-0.99)	1.02 (1.00-1.04)	1.02 (0.96-1.08)	8/14(13)/4	0.010	83.4 (<0.001)	0.032	0.048	0.70-1.22	0.78-1.34	Yes	No	Weak
Mass 2012	Cohort	6	NA/1466	1.00 (0.80-1.19)	1.03 (0.85-1.25)	1.01 (0.89-1.14)	0.99 (0.83-1.18)	1/4/1	0.619	45.6 (0.101)	0.764	0.880	0.60-1.76	0.62-1.65	No	Yes	Non-significant
Bonovas 2008	Overall	19(18)*	62327/884871	0.95 (0.73-1.23)	0.94 (0.72-1.22)	0.61 (0.60-0.63)	0.46 (0.45-0.48)	2/14(13)/3	0.015	98.1 (<0.001)	0.623	<0.001	0.30-2.90	0.20-1.83	Yes	No	Non-significant
Bonovas 2008	RCT	6	1058/40178	1.06 (0.93-1.20)	1.06 (0.93-1.20)	1.06 (0.94-1.19)	1.00 (0.80-1.26)	0/5/1	0.838	9.0 (0.360)	0.386	0.372	0.85-1.31	0.85-1.31	No	Yes	Non-significant
Bonvoas 2008	Observational studies	13(12)*	61314/844693	0.89 (0.65-1.24)	0.88 (0.64-1.21)	0.60 (0.58-0.61)	0.46 (0.45-0.48)	2/9(8)/2	0.080	98.6 (<0.001)	0.432	<0.001	0.26-2.98	0.19-1.91	Yes	No	Non-significant
Taylor 2008	Case-control	7	NA/518043	0.75 (0.50-1.11)	0.74 (0.45-1.20)	0.57 (0.55-0.58)	0.35 (0.20-0.48)	2/4/1	0.504	99.1 (<0.001)	0.216	<0.001	0.14-3.93	0.12-2.66	No	No	Non-significant
**Non-melanoma skin cancer**	Overall	17	NA/1240281	-	1.07 (1.00-1.16)	1.09 (1.06-1.12)	1.09 (1.06-1.13)	1/11/5	0.768	58.5 (0.001)	0.063	<0.001	0.88-1.31	0.90-1.32	No	No	Non-significant
Yang 2017	Overall	12	57004/1591372	-	1.10 (1.01-1.20)	1.10 (1,07-1.13)	1.09 (1.06-1.13)	1/6/5	0.876	66.6 (0.001)	0.031	<0.001	0.87-1.38	0.89-1.36	No	Yes	Weak
Yang 2017	RCT	7	1211/63157	1.09 (0.85-1.39)	1.09 (0.85-1.39)	1.05 (0.94-1.17)	1.03 (0.87-1.22)	1/4/2	0.679	59.9 (0.021)	0.510	0.384	0.58-2.04	0.60-1.83	No	Yes	Non-significant
Yang 2017	Observational studies	5	55793/1528215	1.11 (1.02-1.22)	1.11 (1.02-1.22)	1.10 (1.07-1.13)	1.09 (1.06-1.13)	0/2/3	0.840	76.8 (0.002)	0.015	<0.001	0.82-1.50	0.84-1.44	No	Yes	Weak
Li 2014	Overall	10	3578/103260	1.03 (0.90-1.19)	1.03 (0.90-1.19)	1.12 (1.05-1.20)	1.62 (0.66-3.88)	1/8/1	0.158	61.7 (0.007)	0.651	0.001	0.70-1.52	0.79-1.61	No	Yes	Non-significant

D/N/I=Decreasing risk/No difference/Increasing risk; ES=Effect size; CI=Confidence interval; PI, Prediction interval

§ Risk ratio (95% Confidence interval) of the largest study in each meta-analysis.

† $\mathbf{I^2}$ metric of inconsistency (95% confidence interval of $\mathbf{I^2}$) and P-value of the Cochran Q test for evaluation of heterogeneity.

* Studies with asymmetric effect size are excluded from re-analysis.

** Convincing or suggestive level of evidence due to the greater number of studies that decrease risk.

Supplementary Table S2. PRISMA checklist

Section/topic	#	Checklist item	Reported on page #
**TITLE**			
Title	1	Identify the report as a systematic review, meta-analysis, or both.	1
**ABSTRACT**			
Structured summary	2	Provide a structured summary including, as applicable: background; objectives; data sources; study eligibility criteria, participants, and interventions; study appraisal and synthesis methods; results; limitations; conclusions and implications of key findings; systematic review registration number.	1
**INTRODUCTION**			
Rationale	3	Describe the rationale for the review in the context of what is already known.	2
Objectives	4	Provide an explicit statement of questions being addressed with reference to participants, interventions, comparisons, outcomes, and study design (PICOS).	2
**METHODS**			
Protocol and registration	5	Indicate if a review protocol exists, if and where it can be accessed (e.g., Web address), and, if available, provide registration information including registration number.	NA
Eligibility criteria	6	Specify study characteristics (e.g., PICOS, length of follow-up) and report characteristics (e.g., years considered, language, publication status) used as criteria for eligibility, giving rationale.	2-3
Information sources	7	Describe all information sources (e.g., databases with dates of coverage, contact with study authors to identify additional studies) in the search and date last searched.	2
Search	8	Present full electronic search strategy for at least one database, including any limits used, such that it could be repeated.	2-3
Study selection	9	State the process for selecting studies (i.e., screening, eligibility, included in systematic review, and, if applicable, included in the meta-analysis).	2-3, Figure 1
Data collection process	10	Describe method of data extraction from reports (e.g., piloted forms, independently, in duplicate) and any processes for obtaining and confirming data from investigators.	2-4
Data items	11	List and define all variables for which data were sought (e.g., PICOS, funding sources) and any assumptions and simplifications made.	2-4

Risk of bias in individual studies	12	Describe methods used for assessing risk of bias of individual studies (including specification of whether this was done at the study or outcome level), and how this information is to be used in any data synthesis.	NA
Summary measures	13	State the principal summary measures (e.g., risk ratio, difference in means).	3-4
Synthesis of results	14	Describe the methods of handling data and combining results of studies, if done, including measures of consistency (e.g., I^2) for each meta-analysis.	3-4

Risk of bias across studies	15	Specify any assessment of risk of bias that may affect the cumulative evidence (e.g., publication bias, selective reporting within studies).	NA
Additional analyses	16	Describe methods of additional analyses (e.g., sensitivity or subgroup analyses, meta-regression), if done, indicating which were pre-specified.	NA
**RESULTS**			
Study selection	17	Give numbers of studies screened, assessed for eligibility, and included in the review, with reasons for exclusions at each stage, ideally with a flow diagram.	4,<br>Figure 1
Study characteristics	18	For each study, present characteristics for which data were extracted (e.g., study size, PICOS, follow-up period) and provide the citations.	4,<br>Figure 1
Risk of bias within studies	19	Present data on risk of bias of each study and, if available, any outcome level assessment (see item 12).	N/A
Results of individual studies	20	For all outcomes considered (benefits or harms), present, for each study: (a) simple summary data for each intervention group (b) effect estimates and confidence intervals, ideally with a forest plot.	6-9,<br>Supplements
Synthesis of results	21	Present results of each meta-analysis done, including confidence intervals and measures of consistency.	6-9,<br>Table 1-4
Risk of bias across studies	22	Present results of any assessment of risk of bias across studies (see Item 15).	6-9,<br>Table 1-4,<br>Supplements
Additional analysis	23	Give results of additional analyses, if done (e.g., sensitivity or subgroup analyses, meta-regression [see Item 16]).	N/A
**DISCUSSION**			
Summary of evidence	24	Summarize the main findings including the strength of evidence for each main outcome; consider their relevance to key groups (e.g., healthcare providers, users, and policy makers).	10-12
Limitations	25	Discuss limitations at study and outcome level (e.g., risk of bias), and at review-level (e.g., incomplete retrieval of identified research, reporting bias).	12

Conclusions	26	Provide a general interpretation of the results in the context of other evidence, and implications for future research.	12-13
**FUNDING**			
Funding	27	Describe sources of funding for the systematic review and other support (e.g., supply of data); role of funders for the systematic review.	13

From: Moher D, Liberati A, Tetzlaff J, Altman DG, The PRISMA Group (2009). Preferred Reporting Items for Systematic Reviews and Meta-Analyses: The PRISMA Statement. PLoS Med 6(7): e1000097. doi:10.1371/journal.pmed1000097

For more information, visit: **www.prisma-statement.org**.
